# Molecular basis of permethrin and DDT resistance in an *Anopheles funestus* population from Benin

**DOI:** 10.1186/s13071-018-3115-y

**Published:** 2018-11-20

**Authors:** Genevieve Tchigossou, Rousseau Djouaka, Romaric Akoton, Jacob M Riveron, Helen Irving, Seun Atoyebi, Kabirou Moutairou, Akadiri Yessoufou, Charles S Wondji

**Affiliations:** 1 International Institute of Tropical Agriculture, Cotonou, 08 BP 0932 Benin; 2 University of Abomey Calavi, BP 526 Cotonou, Benin; 3 Liverpool School of Tropical Medicine, Pembroke Place, Liverpool, L3 5QA UK; 4 Cell Biology and Genetics Unit, Department of Zoology, University of Ibadan, Ibadan, Oyo State Nigeria; 5 LSTM Research Unit at the Centre for Research in Infectious Diseases (CRID), P.O. Box 13591, Yaoundé, Cameroon

**Keywords:** *Anopheles funestus*, Insecticide resistance, Permethrin, DDT, Kpome, Resistance mechanisms

## Abstract

**Background:**

Insecticide resistance in *Anopheles* mosquitoes is threatening the success of malaria control programmes. In order to implement suitable insecticide resistance management strategies, it is necessary to understand the underlying mechanisms involved. To achieve this, the molecular basis of permethrin and DDT resistance in the principal malaria vector, *Anopheles funestus* from inland Benin (Kpome), was investigated.

**Results:**

Here, using a microarray-based genome-wide transcription and qRT-PCR analysis, we showed that metabolic resistance mechanisms through over-expression of cytochrome P450 and glutathione S-transferase genes (GSTs) are a major contributor to DDT and permethrin resistance in *Anopheles funestus* from Kpome. The *GSTe2* gene was the most upregulated detoxification gene in both DDT- [fold-change (FC: 16.0)] and permethrin-resistant (FC: 18.1) mosquitoes suggesting that upregulation of this gene could contribute to DDT resistance and cross-resistance to permethrin. *CYP6P9a* and *CYP6P9b* genes that have been previously associated with pyrethroid resistance were also significantly overexpressed with FC 5.4 and 4.8, respectively, in a permethrin resistant population. Noticeably, the GSTs, *GSTd1-5* and *GSTd3*, were more upregulated in DDT-resistant than in permethrin-resistant *Anopheles funestus* suggesting these genes are more implicated in DDT resistance. The absence of the L1014F or L1014S *kdr* mutations in the voltage-gated sodium channel gene coupled with the lack of directional selection at the gene further supported that knockdown resistance plays little role in this resistance.

**Conclusions:**

The major role played by metabolic resistance to pyrethroids in this *An. funestus* population in Benin suggests that using novel control tools combining the P450 synergist piperonyl butoxide (PBO), such as PBO-based bednets, could help manage the growing pyrethroid resistance in this malaria vector in Benin.

**Electronic supplementary material:**

The online version of this article (10.1186/s13071-018-3115-y) contains supplementary material, which is available to authorized users.

## Background

There were an estimated 216 million cases of malaria worldwide in 2016 and 445,000 deaths with 80% of all malaria deaths occurring in Africa [1]. Despite extensive control efforts, over half the world’s population remains at risk and the disease has a massive impact on health and economic development, particularly in Africa [2]. Four species of *Anopheles*, *An. gambiae* Giles, *An. coluzzii* Coetzee & Wilkerson, *An. arabiensis* Patton and *An. funestus* Giles, are responsible for most of the malaria transmission in this continent. The transmission role of *An. funestus* (*sensu stricto*) is further supported by observations that in many regions of Africa, its infection rate even surpasses that of *An. gambiae* [3]. Long-lasting insecticidal nets (LLINs) and indoor residual spraying (IRS) are the main malaria prevention interventions [4]. However, the success of these control methods is jeopardised by the development of resistance by *Anopheles* species to insecticides such as pyrethroids and DDT as seen in the past with loss of efficacy of dieldrin for IRS in west/central Africa [5, 6]. In Benin, due to vector resistance to pyrethroids across the country [7–10], two insecticides of two different classes were used in rotation for IRS: bendiocarb (a carbamate) and pirimiphos-methyl (an organophosphate) [11]. However, DDT is still retained for use in IRS, due to the limited number of cost-effective alternatives [12]. Field studies on insecticide susceptibility carried out as baseline surveys for malaria control programs showed *An. funestus* to be resistant to various insecticides at various localities [9, 10, 13]. Metabolic resistance is the main resistance mechanism recorded, and cytochrome P450 genes are playing a major role while target-site resistance like the knockdown resistance (kdr) is absent [9, 14–17]. Target site resistance was investigated in the pyrethroid/DDT-resistant population and the fifth and sixth segments of domain II in the sodium channel sequence and no polymorphism associated with resistance was identified [9, 14, 16, 18–20] although some mutations have been detected in Cameroon [21] and Uganda [22] but with no association yet established with resistance phenotype. A significant increase in mono-oxygenase activities confers resistance to pyrethroids in *An. funestus* in Mozambique and South-Africa (southern Africa) [15, 23–25], Uganda (east Africa) [16, 22, 26], western Kenya (east Africa) [18, 22], Cameroon (central Africa) [21] and in Senegal (west Africa) [19]. Characterisation of the molecular basis of resistance to pyrethroids and DDT in *An. funestus* from the coastal area of Benin [27] revealed a predominant role played by the glutathione S-transferase *GSTe2*. The enzyme encoded by this gene was highly upregulated in association with the presence of an L119F mutation in the substrate binding site conferring a high level of DDT and permethrin resistance [27]. Recently, *An. funestus* from Kpome, an inland region of Benin has also been shown to be resistant to DDT and permethrin with mortality rates of 9.1 ± 2.5% and 13.0 ± 3%, respectively [10]. However, it remains unknown if the resistant mechanism is the same as observed in Pahou, the coastal area of Benin [9] or whether different resistance mechanisms are responsible for the spread of resistance across the country. Such information is essential in designing suitable control interventions nationwide and to improve the implementation of resistance management strategies against *An. funestus*. In this study, using a genome-wide microarray-based transcription analysis, the underlying molecular mechanisms conferring DDT and permethrin resistance in Kpome were characterised revealing that both glutathione S-transferases, notably the *GSTe2*, and cytochrome P450 genes are the main drivers of resistance.

## Methods

### Area of study and mosquito collection

Blood-fed adult female *An. funestus* mosquitoes resting indoors were collected in houses between 06:00 and 10:00 h from the rural area of Kpome (6°23'N, 2°13'E) a southern inland region of Benin from December 2013 to February 2014. Kpome is a large agricultural setting with the intensive production of tomatoes which mainly serve a commercial purpose and insecticides, mainly pyrethroids (deltamethrin and lambda-cyalothrin), used in public health are also used to protect tomatoes against pest attacks. Mosquito collections and rearing were performed as described previously [10]. Adult female mosquitoes were collected indoor between 06:00 to 10:00 h using electric aspirators. F1 adults were generated from field-collected female mosquitoes using the forced-egg laying method [16] and were randomly mixed in cages for subsequent experiments. All females used for individual oviposition were morphologically and molecularly identified as *An. funestus* (*s.s.*) [10, 28].

### Insecticide susceptibility assays

Two to five day-old F1 adult female mosquitoes pooled from different F0 mosquitoes collected from Kpome were used for this test. Twenty to twenty-five mosquitoes per tube with at least four replicates were exposed to DDT (4%) and permethrin (0.75%) for 1 h before transferring into clean holding tube with 10% sugar solution. Final mortality was scored 24 h post-exposure. Survivors and dead mosquitoes were conserved for further analysis. In this study, we used the same mosquitoes from the previously published research results [10] to further describe the molecular basis of the observed resistance in this malaria vector population. Mosquito samples generated from the previous investigation were used for genetic analysis in this work. The susceptible strain used in this study has the same age as the exposed mosquitoes.

### Microarrays

A genome-wide transcription profiling was performed to detect the sets of genes differentially expressed in relation to observed resistance phenotypes in *An. funestus* populations from Kpome. The microarray hybridisation for *An. funestus* was carried out using the 8 × 60 k (60 mer) Agilent *An. funestus* chip. This Agilent microarray chip was designed using the eArray program (Agilent, Santa Clara, CA, US ) (A-MEXP-2374) by adding the 15,527 expressed sequence tags (ESTs) generated from another transcriptome sequencing of *An. funestus* [29] to the previous 4 × 44 k array (A-MEXP-2245) [24]. Each array was designed using 8540 ESTs generated from *An. funestus* transcriptome 454 sequencing [30], 2850 *An. funestus* cDNAs from GenBank and a set of P450 genes from the *rp1* and *rp2* QTL BAC sequence [31, 32], and the 13,000 transcripts of the complete *An. gambiae* genome. Moreover, *An. gambiae* detoxification genes previously present on the *An. gambiae* detox chip [33] were added to this. RNA was extracted from three batches of 10 *An. funestus* females (2–5 days-old) from the following sample sets: alive after exposure to 0.75% permethrin (Resistant permethrin: R_perm_) and 4% DDT (Resistant DDT: R_DDT_), un-exposed to insecticides (Control: C), and from the fully susceptible laboratory strain FANG (Susceptible: S) [a strain originating from Calueque in southern Angola in 2002 which is completely susceptible to insecticides (Hunt et al. [34]) using the Picopure RNA Isolation Kit (Arcturus, Waltham, MA, USA). The FANG colony was kept in insectary conditions (temperature of 25–28 °C with a relative humidity of 80%) at the Liverpool School of Tropical Medicine where assays were performed. RNA quantity and quality were assessed using a NanoDrop ND1000 spectrophotometer (Thermo Fisher Scientific, Waltham, MA, USA) and Bioanalyzer (Agilent, Santa Clara, CA, USA), respectively. Each RNA sample was used to generate complementary RNA (cRNA) using the Agilent Quick Amp Labeling Kit (two-colour) following the manufacturer’s protocol. cRNAs from the resistant samples (R) were labelled with a cy5 dye, and cRNAs from the susceptible strain FANG (S) were labelled with the cy3 dye. cRNA quantity and quality were assessed using the NanoDrop and Bioanalyzer before labelling. Labelled cRNAs were hybridised to the arrays for 17 h at 65 °C according to the manufacturer’s protocol. Five hybridisations were performed for each comparison including two dyes swap per comparison. Microarray data were analysed using Genespring GX 13.0 software purchased from Agilent (Agilent). A normalisation of the data was performed with the GeneSpring feature extraction program using the lowess normalisation approach. To identify differentially expressed genes, a cut-off of 2-fold-change (FC) and a statistical significance of *P* ≤ 0.05 with Benjamini-Hochberg correction for multiple testing and Storey with bootstrapping (with a cut-off of 1.5-fold-change for the R-C comparison) were applied. A false discovery analysis was also performed using the Benjamini-Hochberg correction test for multiple testing as implemented in GeneSpring.

### Quantitative reverse transcriptase PCR

A qRT-PCR was used to validate the microarrays results for seven of some upregulated detoxification genes. These genes include three cytochromes P450s (*CYP6M7, CYP6P9a, CYP9K1*), two glutathione transferases (*GSTd1-5, GSTe2*), and one aldehyde oxidase (*Ald oxi*). Also, the expression level of *CYP6P9b* previously found to be strongly associated with pyrethroid resistance [19, 31, 35] and *GSTd1-3*, a gene shown as possibly associated with pyrethroid resistance by 454 transcriptome profiling [19, 30] were analysed (Table 1). One microgram of the three biological replicates for resistant (permethrin: R_perm_ and DDT: R_DDT_), control (C), and FANG (S) was used for cDNA synthesis using Superscript III (Invitrogen, Carlsbad, CA, USA) with oligodT20 and RNase H according to the manufacturer’s instructions. Standard curves were established for each gene using serial dilution of cDNA. qRT-PCR amplification was performed using the MX 3005P (Agilent) system. The relative expression level and fold change (FC) of each target gene in resistant and control samples relative to susceptible were calculated according to the 2-ΔΔCT method incorporating the PCR efficiency [36] after normalisation with the housekeeping genes *ribosomal protein S7* (*RSP7*; AFUN007153-RA), and *actin* (*Act5C*; AFUN006819).

**Table 1:** List of primers used for qRT-PCR

Primers	Forward	Reverse	Expected size (bp)
*CYP6M7*	CCAGATACTGAAAGAGAGCCTTCG	CAAGCACTGTCTTCGTACCG	102
*CYP6P9a*	CAGCGCGTACACCAGATTGTGTAA	TCACAATTTTTCCACCTTCAAGTAATTACCCGC	92
*CYP6P9b*	CAGCGCGTACACCAGATTGTGTAA	TTACACCTTTTCTACCTTCAAGTAATTACCCGC	97
*GSTe2*	GTTTGAAGCAGTTGCCATACTACGAGG	TCAAGCTTTAGCATTTTCCTCCTTTTTGGC	101
*GSTd3*	CACGGCCAGTCCTCTTTTAG	AAGCTTCTTCGCCACCAGTA	128
*GSTd1-5*	TGGAGAAATACGGCAAGGAC	CTTGGCGAAGATTTGTGGAT	140
*Aldehyde oxidase*	GCTCTGAACATTGCACCTCA	TGGTGTCGAACGATTGTGTT	109
*CYP9K1*	AGGGCTTCTGGATACGGTTC	CGTACGGTTCGGTTTTGATT	103
*RSP7*	GTGTTCGGTTCCAAGGTGAT	TCCGAGTTCATTTCCAGCTC	98
*Actin*	TTAAACCCAAAAGCCAATCG	ACCGGATGCATACAGTGACA	111

### Investigation of the role of the knockdown resistance mutation in DDT and permethrin resistance

A fragment spanning a portion of the voltage-gated sodium channel gene (*VGSC*), including the 1014 codon (a portion of intron 19 and the entire exon 20, domain II, segment 6 of the *VGSC* gene) associated with resistance in *An. gambiae*, was amplified in eleven field-collected females of *An. funestus* from Kpome using the Exon 19F/KdrFunR2 primers (Exon 19F: 5'-TTT TTA AGC TCG CTA AAT CGT G-3'; KdrFunR2: 5'-CCG AAA TTT GAC AAA AGC AAA-3') [9, 15, 22]. The PCR products were purified using the QIAquick Purification Kit (Qiagen, Hilden, Germany) and directly sequenced. The polymorphic positions were detected through manual analysis of sequence traces using BioEdit. Sequences were aligned using ClustalW [37], and haplotype reconstruction and analysis were done using DnaSP v5.10 [38]. Kpome sequences were also compared to those previously obtained from Pahou in coastal Benin [9] and Gounougou in North Cameroon [21]. After the selection of the best model, a maximum likelihood phylogenetic tree was generated for the VGSC haplotypes in the different populations using Mega 6.06 [39]. The level of *Kst* of pairwise genetic differentiation between the populations was estimated in Dnasp v5.10, and the neighbour-Joining tree was built using Mega 6.06.

## Results

### Genome-wide microarray-based transcriptional profiling of permethrin resistance

**Fig. 1:**
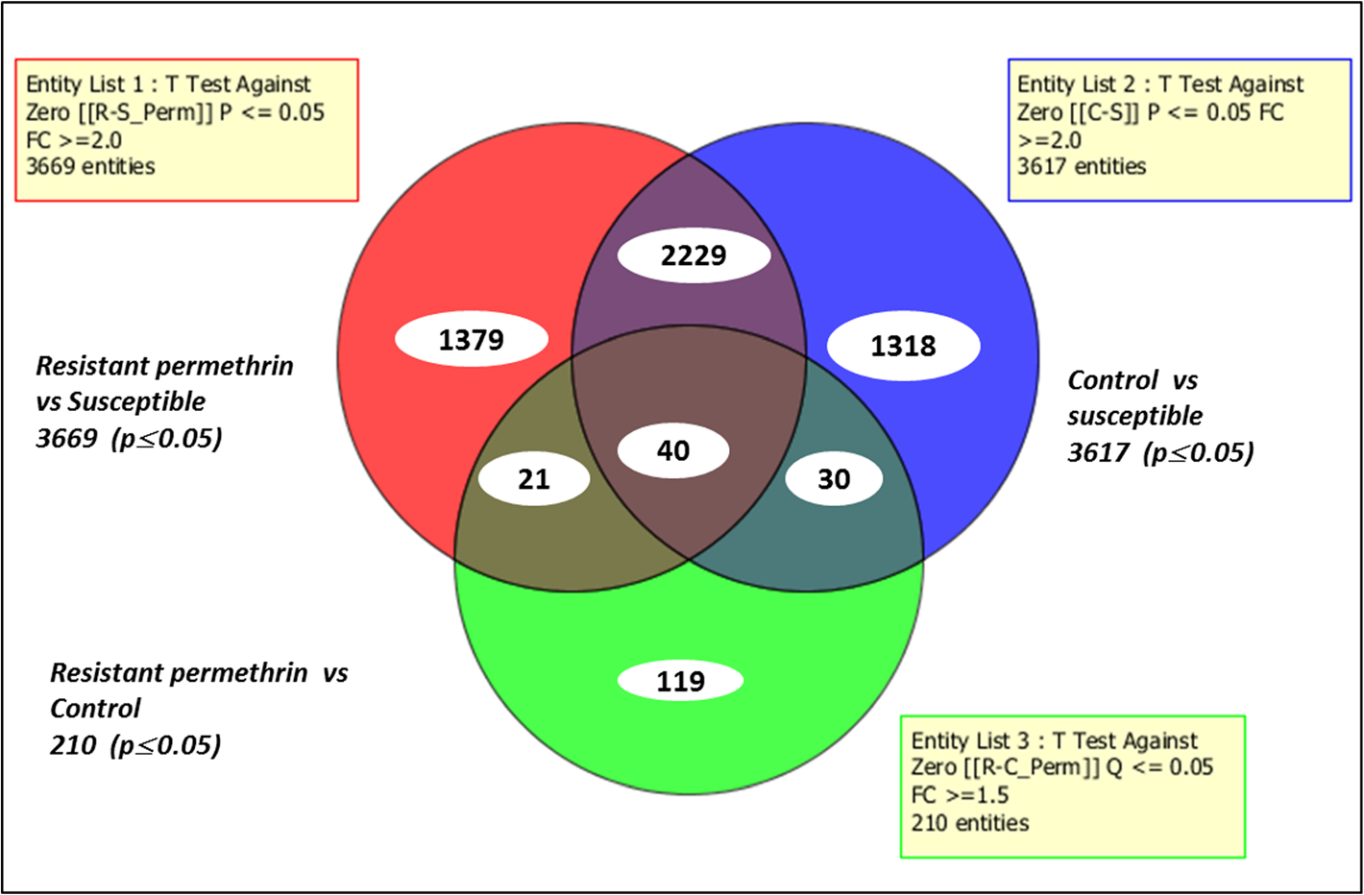
Summary of transcripts differentially expressed in permethrin resistance. The Venn diagrams show the number of transcripts significantly (*P* ≤ 0.05) up- or downregulated (FC ≥ 2) in each comparison as well as the commonly expressed transcripts

A set of transcripts were differentially expressed (≥ 2-fold change, *P* ≤ 0.05) between permethrin resistant (R_perm_), susceptible (S) and control (C) mosquitoes (Fig. 1) from microarray analysis. Overall, 3669 transcripts were differentially expressed when permethrin resistant mosquitoes were compared to susceptible strains (1460 overexpressed and 2209 underexpressed), 3617 between C-S (1211 overexpressed and 2406 underexpressed) and 210 for R_perm_-C (91 overexpressed and 119 underexpressed). Overall, a total of 40 transcripts were differentially expressed in all the three comparisons.

### Genes commonly overexpressed in R_perm_-S, C-S and R_perm_-C comparisons

Only the transcript, AGAP011496-PA (*Anopheles gambiae* str. PEST) was upregulated in R_perm_-S, R_perm_-C and C-S comparisons with FC of 2.7, 1.5 and 2.3, respectively. No detoxification gene was found in the comparison of the three groups of mosquitoes.

### Genes commonly overexpressed in R_perm_-S and C-S comparisons

Several detoxification genes were commonly over-expressed in the R_perm_-S and C-S. Among these genes, the transcripts for *glycogenin* (Afun000500) and *CYP6M7*, belonging to the cytochrome P450 gene family, were the most commonly overexpressed detoxification gene in R_perm_-S (FC of 36.2 for Afun000500 and 34.6 for *CYP6M7*) and C-S (FC of 38.1 for Afun000500 and 28.9 for *CYP6M7*). A transcript from *CYP6AA1* was also commonly upregulated in the R_perm_-S and C-S samples with FC of 5.2 and 3.8, respectively. This gene is the ortholog of *CYP6AA3* in *An. minimus*, which was shown to metabolise pyrethroids [40]. Other upregulated detoxification genes identified in R_perm_-S and C-S comparisons include *CYP6Y2* located in the genomic region spanning the pyrethroid resistance *rp2* QTL detected in the FUMOZ-R pyrethroid-resistant laboratory strain [32], as well as *CYP6M4*, *CYP6S1*, *CYP4K2* and *CYP315A1* that were expressed at lower levels (FC < 5) (Table 2). Transcripts from the glutathione S-transferase (GST) family notably from epsilon [*GSTe2* (FC of 10.9 in R_perm_-S and 15.2 in C-S) and *GSTe4* (FC of 3.9 in R_perm_-S and 4.7 in C-S)] and delta, *GSTd1-5* (FC 2.0 and 2.6 in R_perm_-S and C-S, respectively) classes were upregulated. Other genes such as *ABC transporter* (Afun009697), *alcohol dehydrogenases* (Afun012461*,* Afun013797), *carboxylesterase* (Afun009492), *short chain dehydrogenase* (combined_c738) and *chymotrypsin* (Afun013921) were also overexpressed (Table 2 and Additional file 1: Table S1).

**Table 2:** Top detoxification genes upregulated in Rperm-S, C-S and Rperm-C

Probe name	Systematic name	Rperm-S fold change (FC)	C-S fold change (FC)	Rperm-C fold change (FC)	Ortholog in *An. gambiae*	Description
CUST_5822_PI426302897	Afun005822	2.7	2.31	1.5	AGAP011496-PA	AGAP011496-PA [*Anopheles gambiae* str. PEST]
CUST_500_PI426302897	Afun000500	36.2	38.1		na	Glycogenin
CUST_7663_PI426302897	Afun007663 (CYP6M7)	34.6	28.9		AGAP008213-PA	Cytochrome p450 6a8
CUST_8887_PI426302897	Afun008887	34.0	16.8		AGAP011997-PA	Nucleotide binding protein 2
CUST_9227_PI426302897	Afun009227	23.3	25.6		AGAP008141-PA	Argininosuccinate lyase
CUST_1459_PI406199769	combined_c738	22.0	34.5			Short-chain dehydrogenase
CUST_13921_PI426302897	Afun013921	19.1	21.1		AGAP006709-PA	Chymotrypsin 1
CUST_9492_PI426302897	Afun009492	16.4	3.7		AGAP001722-PA	Carboxylesterase
CUST_1822_PI406199769	combined_c920	11.1	11.4			Glutathione-S-transferase gst
CUST_45_PI426302897	Afun000045 (GSTE2)	10.9	15.2		AGAP009194-PA	Glutathione-S-transferase gst
CUST_4223_PI426302897	Afun004223	10.6	9.4		AGAP008358-PA	Cytochrome p450 4d1
CUST_12461_PI426302897	Afun012461	8.4	10.9		AGAP000288-PA	Alcohol dehydrogenase
CUST_3220_PI426302897	Afun003220	5.7	7.0		AGAP002867-PA	Cytochrome p450
CUST_8615_PI426302897	Afun008615 (CYP6AA1)	5.2	3.8		AGAP002862-PA	Cytochrome p450
CUST_9697_PI426302897	Afun009697	4.8	6.8		AGAP006364-PA	Abc transporter
CUST_9_PI426302915	CYP6M4.seq	4.8	3.5			Cytochrome p450
CUST_13797_PI426302897	Afun013797	4.1	3.0		AGAP000289-PA	Alcohol dehydrogenase
CUST_8445_PI426302897	Afun008445 (GSTE4)	3.9	4.7		AGAP009193-PA	Glutathione-S-transferase gst
CUST_25_PI426302915	CYP6Y2_rvcpl.seq	3.7	3.8			Cytochrome p450
CUST_1392_PI426302897	Afun001392	3.3	3.4		na	Glycine dehydrogenase
CUST_3394_PI426302897	Afun003394 (CYP315A1)	3.1	2.4		AGAP000284-PA	Cytochrome p450
CUST_8909_PI426302897	Afun008909 (CYP4K2)	2.4	2.0		AGAP002416-PA	Cytochrome p450
CUST_20_PI426302915	CYP6S1.seq	2.3	2.2			Cytochrome p450
CUST_7499_PI426302897	Afun007499 (GSTD1)	2.0	2.6		AGAP004164-PA	Glutathione transferase
CUST_11942_PI426302897	Afun011942	6.1			AGAP011509-PA	Carboxylesterase
CUST_4048_PI406199772	CD577343.1	5.3				Cuticle protein
CUST_12343_PI426302897	Afun012343 (CYP4H18)	5.0			AGAP008358-PA	Cytochrome p450 4d1
CUST_10836_PI426302897	Afun010836	4.8			AGAP006228-PA	Esterase b1
CUST_11042_PI426302897	Afun011042	3.9			AGAP003321-PA	Glycine dehydrogenase
CUST_11037_PI426302897	Afun011037	3.7			AGAP003581-PA	Alcohol dehydrogenase
CUST_1_PI426302915	CYP6M1a.seq	2.9				Cytochrome p450
CUST_7501_PI406199798	AGAP007662-RA_2L	2.6			AGAP007662-RA_2L	Short-chain dehydrogenase
CUST_9335_PI426302897	Afun009335 (CYP6AG1)	2.4			AGAP003343-PA	Cytochrome p450
CUST_7769_PI426302897	Afun007769 (CYP9K1)	2.2			AGAP000818-PA	Cytochrome p450 cyp9k1
CUST_11899_PI426302897	Afun011899	2.1			AGAP012514-PA	Short-chain dehydrogenase
CUST_4043_PI406199772	CD577345.1		3.7			Cuticle protein
CUST_7722_PI426302897	Afun007722		3.2		AGAP009850-PA	Abc transporter
CUST_12261_PI426302897	Afun012261		2.8		AGAP005758-PA	Carboxylesterase
CUST_13481_PI426302897	Afun013481 (GSTE1)		2.7		AGAP009195-PA	Glutathione-S-transferase gst
CUST_25_PI406199775	CYP6P9a		2.3			Cytochrome p450
CUST_15331_PI426302897	Afun015331 (CYP307A1)		2.2		AGAP001039-PB	Cytochrome p450 307a1
CUST_493_PI426302897	Afun000493		2.1		AGAP006225-PA	Aldehyde oxidase

*Abbreviation*: *na* not available

### Comparative transcriptional profiling of DDT and permethrin resistance

**Fig. 2:**
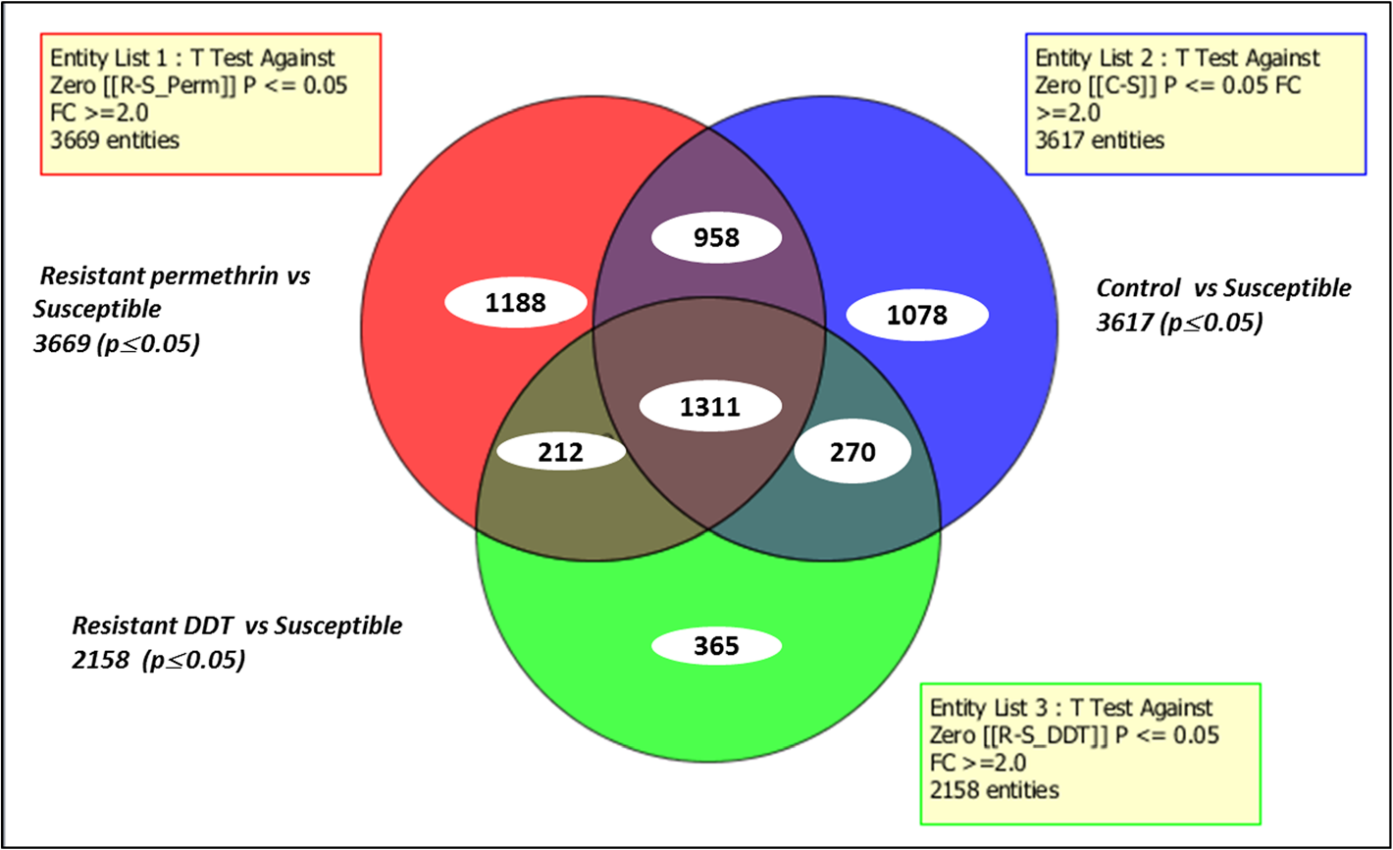
Summary of transcripts differentially expressed in DDT and permethrin resistance. The Venn diagrams show the number of transcripts significantly (*P* ≤ 0.05) up- or downregulated (FC ≥ 2) in each comparison as well as the commonly expressed transcripts permethrin

A comparative analysis of transcriptomes associated with DDT resistance in Kpome showed a total of 2158 transcripts differentially expressed (≥ 2-fold change, FC at *P* ≤ 0.05) in R_DDT_-S with 715 upregulated and 1443 downregulated. The comparison between R_DDT_-S, R_perm_-S and C-S revealed 1311 transcripts differentially expressed. A set of 212 transcripts was commonly expressed between R_DDT_-S and R_perm_-S, 958 transcripts and 270 transcripts were also expressed when comparing R_perm_-S and R_DDT_-S to C-S (Fig. 2).

### Genes commonly overexpressed in R_DDT_-S, Rperm-S and C-S comparisons

Many genes were commonly overexpressed in the three comparisons with *glycogenin* (Afun000500) and *arginosuccinate lyase* (Afun009227) been the most overexpressed and were consistent in all the three comparisons with fold change of (FC 36.2, 38.1 and 41.4) and (FC 22.2, 23.3 and 25.6) in R_DDT_-S, R_perm_-S and C-S, respectively. Among the common detoxification gene families identified, the *GSTe2* gene (Afun000045) was the one that remained highly upregulated with FC of 13.3, 10.9 and 15.2 for R_DDT_-S, R_perm_-S and C-S, respectively. The *CYP6M7* gene was also overexpressed in all three comparisons; FC was 34.6 in R_perm_-S and FC 28.9 in C-S but with lower FC of 4 in R_DDT_-S. Several other genes of the family of P450 such as *CYP6AA1*, *CYP6M4*, *CYP6Y2*, *CYP4K2* and *CYP315A1* were also upregulated. Other transcripts of the GST family were also upregulated such as the *Gste4* (FC 4.3, 3.9 and 4.7 in R_DDT_-S, R_perm_-S and C-S, respectively) and the *GSTd1* (FC 2.2, 2.0 and 2.6 in R_DDT_-S, R_perm_-S and C-S, respectively (Table 3 and Additional file 1: Table S2).

**Table 3:** Top detoxification genes upregulated in R_DDT_-S, Rperm-S and C-S

Probe name	Systematic name	R_DDT_-S fold change (FC)	Rperm-S fold change (FC)	C-S fold change (FC)	Ortholog in *An. gambiae*	Description
CUST_500_PI426302897	Afun000500	36.2	38.1	41.4	na	Glycogenin
CUST_9227_PI426302897	Afun009227	22.2	23.3	25.6	AGAP008141-PA	Argininosuccinate lyase
CUST_45_PI426302897	Afun000045 (GSTE2)	13.3	10.9	15.2	AGAP009194-PA	Glutathione-S-transferase
CUST_4223_PI426302897	Afun004223	12.8	10.6	9.4	AGAP008358-PA	Cytochrome p450 4d1
CUST_9697_PI426302897	Afun009697	11.6	4.8	6.8	AGAP006364-PA	Abc transporter
CUST_1822_PI406199769	combined_c920	11.5	11.1	11.4		Glutathione-S-transferase
CUST_3220_PI426302897	Afun003220	10.8	5.7	7.0	AGAP002867-PA	Cytochrome p450
CUST_9492_PI426302897	Afun009492	7.9	16.4	3.7	AGAP001722-PA	Carboxylesterase
CUST_8615_PI426302897	Afun008615 (CYP6AA1 )	5.1	5.2	3.8	AGAP002862-PA	Cytochrome p450
CUST_9_PI426302915	CYP6M4.seq	4.9	4.8	3.5		Cytochrome p450
CUST_8445_PI426302897	Afun008445 (GSTE4)	4.3	3.9	4.7	AGAP009193-PA	Glutathione-S-transferase
CUST_6930_PI426302897	Afun006930 (CYP6M7)	4.0	34.6	28.9	AGAP008212-PA	Cytochrome p450 6a8
CUST_25_PI426302915	CYP6Y2_rvcpl.seq	3.4	3.7	3.8		Cytochrome p450
CUST_20_PI426302915	CYP6S1.seq	3.0	2.3	2.2		Cytochrome p450
CUST_8909_PI426302897	Afun008909 (CYP4K2)	2.8	2.4	2.0	AGAP002416-PA	Cytochrome p450
CUST_1392_PI426302897	Afun001392	2.5	3.3	3.4	na	Glycine dehydrogenase
CUST_13218_PI426302897	Afun013218 (CYP315A1)	2.3	2.9	2.6	AGAP000284-PA	Cytochrome p450
CUST_7499_PI426302897	Afun007499 (GSTD1)	2.2	2.0	2.6	AGAP004164-PA	Glutathione transferase
CUST_11037_PI426302897	Afun011037	6.5	3.7		AGAP003581-PA	Alcohol dehydrogenase
CUST_12343_PI426302897	Afun012343 (CYP4H18 )	5.2	5.0		AGAP008358-PA	Cytochrome p450 4d1
CUST_10836_PI426302897	Afun010836	4.3	4.8		AGAP006228-PA	Esterase b1
CUST_7769_PI426302897	Afun007769 (CYP9K1 )	3.0	2.2		AGAP000818-PA	Cytochrome p450 cyp9k1
CUST_7469_PI426302897	Afun007469 (CYP9J3)	2.0	2.1		AGAP012296-PA	Cytochrome p450
CUST_12461_PI426302897	Afun012461		8.4	10.9	AGAP000288-PA	Alcohol dehydrogenase
CUST_3489_PI406199769	combined_c1762		2.2	2.2		Abc transporter
CUST_8026_PI426302897	Afun008026		2.1	2.2	AGAP003578-PA	Aldehyde dehydrogenase
CUST_1458_PI406199769	combined_c738	10.4		15.9		Short-chain dehydrogenase
CUST_4043_PI406199772	CD577345.1	4.4		3.7		Cuticle protein
CUST_15331_PI426302897	Afun015331 (CYP307A1)	3.4		2.2	AGAP001039-PB	Cytochrome p450 307a1
CUST_25_PI406199775	CYP6P9a	2.8		2.3		Cytochrome p450
CUST_13481_PI426302897	Afun013481 (GSTE1 )	2.5		2.7	AGAP009195-PA	Glutathione-S-transferase gst
CUST_4048_PI406199772	CD577343.1		5.3			Cuticle protein
CUST_11042_PI426302897	Afun011042		3.9		AGAP003321-PA	Glycine dehydrogenase
CUST_1_PI426302915	CYP6M1a.seq		2.9			Cytochrome p450
CUST_9335_PI426302897	Afun009335 (CYP6AG1)		2.4		AGAP003343-PA	Cytochrome p450
CUST_13475_PI426302897	Afun013475	3.2			AGAP003582-PA	Alcohol dehydrogenase
CUST_5448_PI426302897	Afun005448 (CYP302A1)	2.6			AGAP005992-PA	Cytochrome p450
CUST_4047_PI406199772	CD577343.1	2.4				Cuticle protein
CUST_8823_PI426302897	Afun008823 (CYP4D15 )	2.4			AGAP002418-PA	Cytochrome p450
CUST_7301_PI426302897	Afun007301 (CYP4J5)	2.2			AGAP006048-PA	Cytochrome p450
CUST_208_PI406199788	gb-CYP12F3	2.1				Cytochrome p450
CUST_10630_PI426302897	Afun010630	2.1			AGAP002866-PA	Cytochrome p450
CUST_7722_PI426302897	Afun007722			3.2	AGAP009850-PA	Abc transporter
CUST_12261_PI426302897	Afun012261			2.8	AGAP005758-PA	Carboxylesterase
CUST_4088_PI406199772	CD577323.1			2.4		Cuticle protein
CUST_493_PI426302897	Afun000493			2.1	AGAP006225-PA	Aldehyde oxidase

*Abbreviation*: *na* not available

### Overexpressed genes in the comparison between R_DDT_-S and Rperm-S

A limited number of genes were commonly overexpressed in these two “resistant” comparisons, potentially as a result of induction or greater involvement in resistance than in control samples. The transcript of *alcohol dehydrogenase* (Afun011037) was the most overexpressed in R_DDT_-S, and R_perm_-S comparison with FC 6.5 and 3.7, respectively. Other genes of the P450 family including *CYP4H18*, *CYP9K1*, *CYP9J3*, and the *esterase b1* were also overexpressed (Table 3 and Additional file 1: Table S2).

### Overexpressed genes in the comparison between R_DDT_-S and C-S

The transcript **(**combined_c738) belonging to *the short-chain dehydrogenase* gene was the most commonly upregulated in the R_DDT_-S, and C-S comparison with FC 10.4 and 15.9, respectively. The cytochrome P450 gene *CYP307A1* was also upregulated with FC 3.3 in R_DDT_-S and FC 2.2 in C-S. The *CYP6P9a* gene, reported to be upregulated in southern Africa mosquitoes, was also upregulated with FC 2.8 and 2.3 in R_DDT_-S and C-S, respectively. Other genes with a known association with insecticide resistance were also upregulated in Kpome mosquitoes as shown in Table 3 and Additional file 1: Table S2.

### Overexpressed genes in the comparison between Rperm-S and C-S

Genes identified from these comparisons include the *alcohol dehydrogenase* gene (Afun012461 ortholog of *AGAP000288* in *An. gambiae*) which is consistently upregulated in R_perm_-S (FC8.4) and C-S (FC10.9) but not in R_DDT_-S. Similarly, the *aldehyde dehydrogenase* (Afun008026) and the *ABC transporter* (Combined*_c1762*) were also upregulated in R_perm_-S and C-S exclusively although with lower FC.

### Overexpressed genes in the comparison between DDT resistant mosquitoes and a susceptible strain

Some detoxification genes specific for R_DDT_-S comparison were identified in Kpome. These genes include *alcohol dehydrogenase* Afun013475 (FC 3.2), the most upregulated gene (FC 3.2) as well as *CYP302A1* (FC 2.6), *CYP4D15* (FC 2.4), *CYP4J5* (FC 2.2), *gb-CYP12F3* (FC 2.1) and the cytochrome P450 (Afun010630) (FC 2.1).

### Overexpressed genes in the comparison between permethrin resistant mosquitoes and a susceptible strain

For R_perm_-S comparison, the *ABC transporter* (Afun007722) was the most upregulated gene with a fold change of 3.2. Also, the *carboxylesterase* (Afun012261) (FC 2.8), the cuticle protein (CD5773231) (FC 2.4) and the *aldehyde oxidase* (Afun00493) (FC 2.4) were upregulated.

### Quantitative reverse transcriptase PCR

**Fig. 3:**
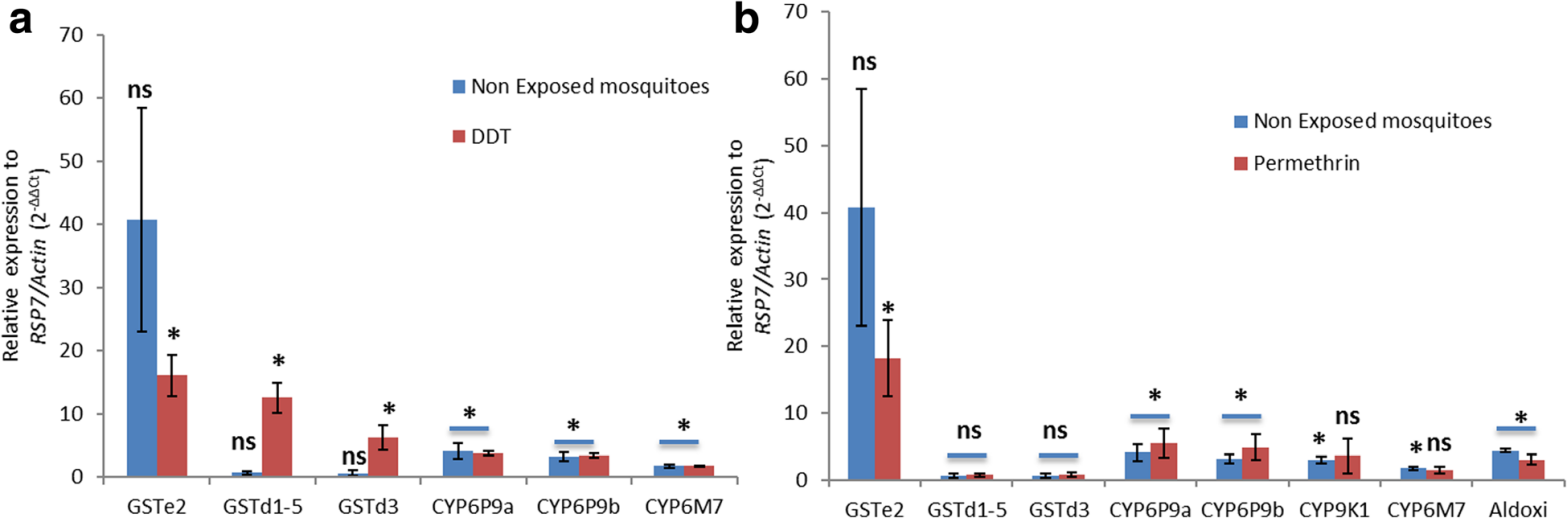
Quantitative PCR results: differential expression by qRT-PCR of some candidate genes upregulated in microarray assays in *An. funestus* resistant to DDT (**a**) and permethrin (**b**). Error bars represent SD (*n* = 3). The presence of * on top of the two-fold changes for each gene indicates a statistically significant (*P* < 0.05) over-expression in resistant or non-exposed mosquitoes compared to the FANG susceptible strain; “ns” is shown when the difference was not significant

A qRT-PCR analysis revealed that *GSTe2* is the most overexpressed genes in DDT and permethrin resistant samples (FC 16.0 and 18.1, respectively) analysed in this study. However, the expression level of the detoxification gene, *CYP6M7* recorded with the qRT-PCR analysis was lower (FC 1.4 and 1.7 in permethrin and DDT resistant samples, respectively) compared to expression level from microarray analysis (FC 34.6 and 4.0 in permethrin and DDT resistant samples, respectively). The *GSTd1-5* and the *GSTd3* were significantly upregulated in DDT resistant samples (FC 12.5 and 6.2; *P* < 0.01) compared to permethrin resistant samples (FC 0.72 and 0.86; *P* < 0.05) (Fig. 3a, b).

### Role of the knockdown resistance gene in DDT and permethrin resistance profiles recorded in *An. funestus* from Kpome

**Fig. 4:**
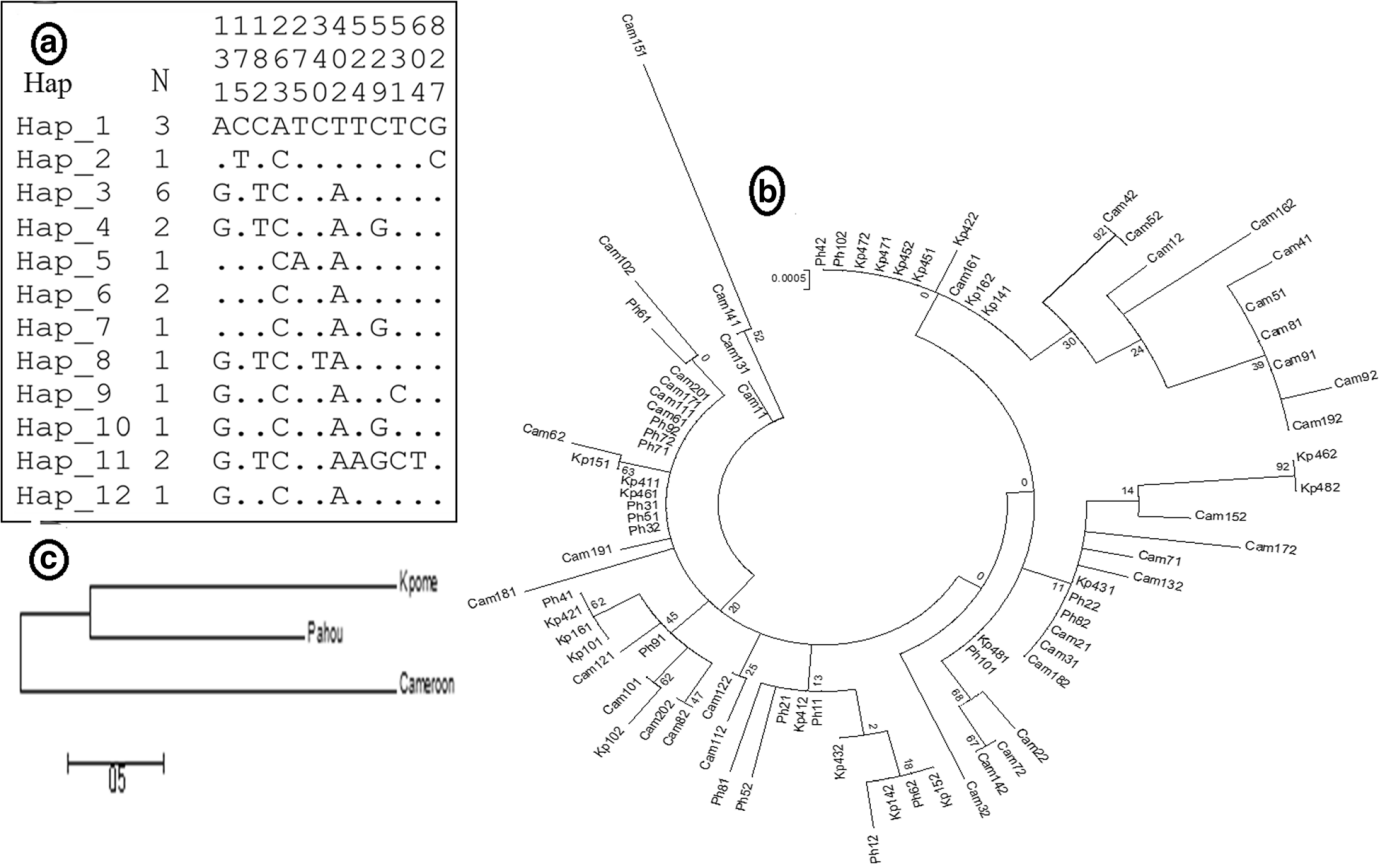
*kdr* polymorphism in *Anopheles funestus* of Kpome. **a** Schematic representation of haplotypes of Exon20 fragment of the voltage-gated sodium channel gene (*VGSC*) observed in wild type *An. funestus* from Kpome. Only polymorphic sites are shown and these are numbered from the beginning of each aligned sequence. Dots indicate identity with the first sequence. A number has been given to each haplotype. The column (N) indicates the number of individuals sharing the haplotype. **b** Maximum-likelihood tree based on *kdr* data for samples from Kpome, Pahou and Cameroon. **c** Neighbour-joining tree based on the *VGSC* gene data for samples of Kpome, Pahou and Cameroon. *Abbreviations*: Kp, Kpome; Ph, Pahou, Cam: Cameroon

Amplification and sequencing of a fragment (a portion of intron 19 and the entire exon 20, domain II, segment 6) of the VGSC gene showed that both L1014F (TTA-to-TTT) and L1014S (TTA-to-TCA) kdr mutation commonly found in *An. gambiae* are absent in this mosquito species. However, further analysis with 837 bp of common sequences generated from the eleven mosquitoes analysed revealed 12 polymorphic sites with 12 haplotypes (Table 4, Fig. 4) showing a high genetic diversity of this gene within the *An. funestus* mosquitoes of Kpome. No amino acid change was detected in the Benin population. Furthermore, the Tajima D and Fu and Li D* statistics (Table 4) were not statistically significant. The Neighbour-joining tree showed low genetic differentiation between Benin populations compared to Cameroon samples with respect to geographical distance (Fig. 4).

**Table 4:** Summary statistics for polymorphism in the voltage-gated sodium channel gene in F_0_ *An. funestus* from Kpome compared to Pahou and Cameroon population

	*N*	S	π	k	h	hd	Syn	Nonsyn	D (*P*-value)	D* (*P*-value)
Kpome	22	12	0.00351	2.93939	12	0.909	0	0	0.37 (*P* > 0.10)	0.32 (*P* > 0.10)
Pahou	20	10	0.00260	2.17895	12	0.905	0	0	0.79 (*P* > 0.10)	0.96 (*P* > 0.10)
Cameroon	40	37	0.00514	4.30128	29	0.977	2	3	1.81 (*P* > 0.10)	2.75 (*P* > 0.10)

*Abbreviations*: *N* number of sequences (2n), *S* number of polymorphic sites, *h* number of haplotypes, *hd* haplotype diversity, *π* nucleotide diversity, *k* mean number of nucleotide differences, *D* Tajima’s test, D* Fu and Li’s test, *Syn* synonymous mutation, *Nonsyn* non synonymous mutation

## Discussion

The WHO Global Plan for Insecticide Resistance Management [12] highlights the necessity to detect and monitor the development of insecticide resistance and characterise the underlying resistance mechanisms to maintain the successes in the reduction of malaria cases across Africa. In this study, we elucidated the molecular basis driving DDT and pyrethroids resistance in an inland *An. funestus* population from Benin.

### Permethrin and DDT resistance in *An. funestus* from Kpome is driven by metabolic resistance

Transcriptional profiling results and the absence of *kdr* mutation points out to a possible metabolic mechanism driving permethrin and DDT resistance in *An. funestus* from Kpome notably through overexpression of genes involved in insecticide detoxification such as cytochrome P450 genes, GSTs, aldehyde oxidases, and other gene families previously associated with resistance of *An. funestus* to insecticides [41].

The cytochrome P450, *CYP6M7* was the most overexpressed detoxification gene in permethrin exposed mosquitoes compared to the FANG susceptible strain as well as to the unexposed mosquitoes from microarrays analysis. However, this consistent information from microarray analysis was not confirmed by qRT-PCR. This discrepancy could be associated with the high genetic redundancy of some P450 genes and a high level of sequence similarity between different genes. These factors could lead to cross amplification contributing to homogenise the expression between samples. It is more likely that *CYP6M7* could also be a key pyrethroid resistance gene in Benin, but further validation is required. New approaches with RNAseq could help to confirm the expression pattern of this gene. On the other hand, only one gene (*AGAP011496-PA*) was commonly expressed in R_perm_-S, C-S and R_perm_-C comparisons. This could be due to the high resistance recorded against permethrin suggesting a less significant difference between the resistant and the control. Such a low number of differentially expressed genes between R-S and C-S comparison is commonly observed in similar studies when the resistance level is high in the population as it is the case here in Kpome [24, 27]. This is because both non-exposed (control; C) and alive mosquitoes after exposure (resistant; R) are all resistant which leads to less difference in the level of gene expression fold change between these two comparisons. The *GSTe2* gene was the most upregulated gene in both DDT and permethrin resistant samples showing that this gene is likely to be involved in both DDT and permethrin resistance as previously shown in coastal Benin [27]. Hence, such genes could confer cross-resistance to both insecticides, and this represents a challenge for the success of the malaria control program. The enzyme encoded by this gene was shown to be able to metabolize both DDT and permethrin [27]. QRT-PCR confirmed the expression level of *GSTe2* in permethrin, DDT resistant and unexposed mosquitoes compared to the susceptible FANG. This observation is in line with the common implication of the *GSTe2* gene in permethrin and DDT resistance in the *An. funestus* population from Pahou (Benin) reported by Riveron et al. [27]. Furthermore, the potential role of *GSTe2* in permethrin resistance observed here has been shown in previous reports that suggested that orthologs of *GSTe2* in other insects are associated with pyrethroids resistance. Indeed, the elevated *GSTe2* expression has been associated with pyrethroid resistance by acting as a pyrethroid-binding protein and sequestering the insecticide [42] or by protecting against oxidative stress and lipid peroxidation induced by pyrethroid exposure [43]. In the yellow fever mosquito *Ae. aegypti*, a partial knockdown of the ortholog of *GSTe2* led to increased mortality to pyrethroids (deltamethrin), indicating that *GSTe2* is also associated with deltamethrin resistance in *Ae. aegypti* [44]. Moreover, the over-expression of *GSTe2* was observed with the high frequency of the 119F resistant allele in Kpome (91% of 119F/F homozygote resistant genotype) [10]. The near fixation of 119F-GSTe2 resistant allele in Kpome, which enlarges the substrate binding sites to increase DDT metabolism [27], coupled with the high overexpression of *GSTe2* highlights the key role played by this gene in DDT resistance as previously observed in Pahou. Orthologs of *GSTe2* have also been shown to be associated with DDT resistance in other mosquito species such as *An. gambiae* and *Ae. aegypti* [33, 44, 45]. Overall, *GSTe2* can confer resistance to DDT and permethrin, and this cross-resistance to pyrethroids is of significant concern for malaria control as *GSTe2* could protect mosquitoes against the major insecticides used to impregnate LLINs in public health. However, the overexpression of several genes from other gene families in this study highlights the complexity of resistance mechanisms suggesting the involvement of other genes than just *GSTe2*. In addition, the transcription profile of the duplicated genes *CYP6P9a* and *CYP6P9b* (which can metabolise both types I and II pyrethroids as shown by Riveron et al. [27]) in pyrethroid resistance in Kpome is different to that observed for *An. funestus* population in southern Africa*.* Indeed these P450 genes were highly upregulated in pyrethroid-resistant laboratory and field population from southern Africa [9, 15, 16, 31, 46, 47] while a lower level of overexpression was recorded in Kpome. The level of expression of *CYP6P9a* and *CYP6P9b* recorded in Kpome *An. funestus* population is similar to what was observed previously in Pahou population (coastal Benin). This result suggests that the two duplicated P450 genes are not strongly associated with permethrin resistance in Benin as observed in Mozambique (southern Africa) [15, 35]. Therefore, the molecular basis of the pyrethroid resistance in Benin is most likely different to that in southern Africa pointing to independent selection events of the pyrethroid resistance across Africa probably under different local selective forces. These different selective pressures could be increased of ITNs coverage across Benin, agricultural use of pesticides [48, 49] and spilt petroleum products [50]. In most African urban areas, insecticides are used for domestic purposes, including the control of mosquitoes in the form of mosquito coils, fumigation bombs or sprays [51]. These insecticides are used in an uncontrolled and heterogeneous manner in term of coverage and doses of insecticides in each household. Such practices may represent an additional selective pressure favouring pyrethroid resistance. The presence of agrochemicals, or industrial pollutants and plant compounds in mosquito breeding sites could also affect insecticide tolerance by modulating mosquito detoxification systems [52]. Concerning DDT resistance, in addition to the *GSTe2* gene, two other detoxification genes of GST family were also upregulated: *GSTd1-5* and *GSTd3*. *GSTd3* was shown to be upregulated in DDT-resistant *An. arabiensis* from an urban site in Burkina Faso [53]. *GSTd1-5* have been previously implicated in coding for enzymes that directly metabolise DDT or have at least been previously associated with the DDT-resistant phenotype [54, 55]. Further validation of the role of these genes in DDT resistance is required. The two duplicated cytochrome P450 genes, *CYP6P9a* (FC = 3.7) and *CYP6P9b* (FC = 3.9), which confer pyrethroid resistance in southern African populations of *An. funestus* [24] were also upregulated in the Benin population. However, because their encoded proteins are unable to metabolise DDT [24], these genes are not likely involved in DDT resistance observed in Kpome mosquitoes. Nevertheless, further investigation is required to validate this hypothesis. Monitoring the insecticide resistance mechanisms that occur within a population should be an essential component to all insecticide-based vector control programs and improving resistance management involves a better understanding of resistance mechanisms. These data contribute to the growing body of knowledge focussed on pyrethroid and DDT resistance in Benin. This situation emphasises the need for natural resistance and vector monitoring so that adjustments to control programmes can be made timeously and accurately.

### Analysis of polymorphisms of the VGSC gene supports a limited role of knockdown resistance

Mechanisms of DDT and permethrin resistance are likely not associated with target site resistance as no kdr mutation was detected in analysed mosquitoes from Kpome. L1014F or L1014S change commonly associated with pyrethroid/DDT resistance in *An. gambiae* was not detected in this population of *An. funestus*, as it was also the case for all populations of this species, analysed so far [9, 19–22]. This suggests that the VGSC is probably evolving neutrally in DDT and permethrin resistance in *An. funestus* population.

Furthermore, the neutrality tests with Tajima D and Fu and Li D* statistics revealed no signature of directional selection on the sodium channel gene suggesting the limited role of knockdown resistance in both DDT and pyrethroid resistance in *An. funestus* in Kpome. The neighbour-joining tree revealed that Kpome mosquitoes cluster with Pahou mosquitoes while they are different to Cameroon mosquitoes. This reveals a reduced gene flow between these populations probably through isolation by distance which can also affect the spread of insecticide resistance genes in this species as previously shown for *An. funestus* populations across the continent [56].

## Conclusions

Metabolic resistance is likely driving resistance to both pyrethroids (permethrin) and DDT in the major malaria vector *An. funestus* in Benin. The glutathione s-transferase gene, *GSTe2* is playing a key role in DDT resistance and most likely is responsible for the observed cross-resistance to pyrethroids in *An. funestus* populations from Kpome and such cross-resistance should be taken into account for the implementation of future insecticide resistance management strategies. Moreover, this study provides knowledge on the resistance profile and underlying resistance mechanisms to the available insecticides in *An. funestus*, a less studied malaria vector in Benin, in order to develop better insecticide resistance diagnostics. Further investigation should be performed on the expression level of target genes to ascertain the role of metabolic mechanisms in DDT and permethrin resistance in this *An. funestus* population. Resistance mechanisms detected in this studied population appear to be different from those identified in other African regions showing the need to characterise mosquito populations at country-level for more appropriate and tailored control interventions.

## Additional file

Additional file 1: **Table S1.** The most upregulated genes in Rperm-S, C-S and Rperm-C. **Table S2.** The most upregulated genes in R_DDT_-S, Rperm-S and C-S comparisons. (DOCX 56 kb)

## Abbreviations

DDT: Dichlorodiphenyltrichloroethane
LSTM: Liverpool School of Tropical Medicine
qRT-PCR: Quantitative reverse transcriptase polymerase chain reaction
VGSC: Voltage-gated sodium channel
WHO: World Health Organisation
