# Supplementary material for: Molecular basis of permethrin and DDT resistance in an Anopheles funestus population from Benin
Source: Parasit Vectors. 2018 Nov 20;11:602. doi: 10.1186/s13071-018-3115-y (PMC6247751; doi:10.1186/s13071-018-3115-y)
Supplement: Supplementary file 1 — Table S1. The most upregulated genes in Rperm-S, C-S and Rperm-C. Table S2. The most upregulated genes in RDDT-S, Rperm-S and C-S comparisons. (DOCX 56 kb) [file 13071_2018_3115_MOESM1_ESM.docx]

**Additional file 1**

**Table S1: The most up-regulated genes in Rperm-S, C-S and Rperm-C**

| **Probe Name** | **Systematic Name** | **Rperm-S** | **C-S** | **Rperm-C** | **Ortholog in *An. gambiae*** | **Description** |  |  |
| --- | --- | --- | --- | --- | --- | --- | --- | --- |
| CUST_5822_PI426302897 | Afun005822 | 2.7 | 2.3 | 1.5 | AGAP011496-PA | AGAP011496-PA [Anopheles gambiae str. PEST] | | |
|  |  |  |  |  |  |  |  |  |
| CUST_10990_PI426302897 | Afun010990 | 150.0 | 122.5 |  | AGAP010693-PA | wd-repeat protein | |  |
| CUST_11932_PI426302897 | Afun011932 | 57.2 | 60.8 |  | AGAP002875-PA | AGAP002875-PA [Anopheles gambiae str. PEST] | | |
| CUST_9217_PI426302897 | Afun009217 | 55.9 | 57.3 |  | AGAP012177-PA | sterile alpha motif domain-containing protein | | |
| CUST_2943_PI426302897 | Afun002943 | 54.3 | 79.3 |  | AGAP010979-PA | NA |  |  |
| CUST_6391_PI426302897 | Afun006391 | 48.9 | 55.7 |  | AGAP010956-PA | AGAP010956-PA [Anopheles gambiae str. PEST] | | |
| CUST_8968_PI426302897 | Afun008968 | 39.8 | 31.4 |  | AGAP011375-PA | selenophosphate synthase | |  |
| CUST_500_PI426302897 | Afun000500 | 36.2 | 38.1 |  | NA | glycogenin |  |  |
| CUST_7663_PI426302897 | Afun007663 (CYP6M7) | 34.6 | 28.9 |  | AGAP008213-PA | cytochrome p450 6a8 | |  |
| CUST_8887_PI426302897 | Afun008887 | 34.0 | 16.8 |  | AGAP011997-PA | nucleotide binding protein 2 (nbp 2) | | |
| CUST_12565_PI426302897 | Afun012565 | 33.7 | 38.0 |  | AGAP011220-PA | AGAP011220-PA [Anopheles gambiae str. PEST] | | |
| CUST_10958_PI426302897 | Afun010958 | 30.5 | 77.9 |  | AGAP012235-PA | prefoldin subunit 6 | |  |
| CUST_2403_PI426302897 | Afun002403 | 28.7 | 33.1 |  | NA | conserved hypothetical protein [Culex quinquefasciatus] | | |
| CUST_3347_PI406199769 | combined_c1691 | 28.5 | 20.7 |  |  | mediator complex | |  |
| CUST_9227_PI426302897 | Afun009227 | 23.3 | 25.6 |  | AGAP008141-PA | argininosuccinate lyase | |  |
| CUST_1459_PI406199769 | combined_c738 | 22.0 | 34.5 |  |  | short-chain dehydrogenase | | |
| CUST_13827_PI426302897 | Afun013827 | 21.9 | 24.9 |  | AGAP013066-PA | conserved hypothetical protein [Culex quinquefasciatus] | | |
| CUST_3346_PI406199769 | combined_c1691 | 20.4 | 20.4 |  |  | mediator complex | |  |
| CUST_13921_PI426302897 | Afun013921 | 19.1 | 21.1 |  | AGAP006709-PA | chymotrypsin 1 | |  |
| CUST_14204_PI426302897 | Afun014204 | 19.1 | 14.1 |  | AGAP004911-PA | AGAP004911-PA [Anopheles gambiae str. PEST] | | |
| CUST_4529_PI426302897 | Afun004529 | 18.6 | 22.1 |  | AGAP012173-PA | ankyrin repeat domain protein | | |
| CUST_9492_PI426302897 | Afun009492 | 16.4 | 3.7 |  | AGAP001722-PA | carboxylesterase | |  |
| CUST_2808_PI426302897 | Afun002808 | 16.1 | 26.0 |  | AGAP000475-PA | btb poz domain-containing protein 3 | | |
| CUST_1894_PI426302897 | Afun001894 | 14.4 | 4.7 |  | AGAP000154-PA | amp dependent ligase | |  |
| CUST_2184_PI426302897 | Afun002184 | 12.4 | 13.3 |  | AGAP008354-PA | tyrosine protein kinase | |  |
| CUST_1822_PI406199769 | combined_c920 | 11.1 | 11.4 |  |  | glutathione-s-transferase gst | | |
| CUST_45_PI426302897 | Afun000045 (GSTE2) | 10.9 | 15.2 |  | AGAP009194-PA | glutathione-s-transferase gst | | |
| CUST_8492_PI406199769 | combined_c4300 | 10.8 | 7.3 |  |  | ---NA--- |  |  |
| CUST_4223_PI426302897 | Afun004223 | 10.6 | 9.4 |  | AGAP008358-PA | cytochrome p450 4d1 | |  |
| CUST_12461_PI426302897 | Afun012461 | 8.4 | 10.9 |  | AGAP000288-PA | alcohol dehydrogenase | |  |
| CUST_10777_PI426302897 | Afun010777 | 6.5 | 4.6 |  | AGAP004091-PA | 28s ribosomal protein s5 | |  |
| CUST_1635_PI406199772 | EE589498.1 | 5.8 | 7.3 |  |  | sg2a salivary protein | |  |
| CUST_5545_PI426302897 | Afun005545 | 5.7 | 4.9 |  | AGAP012173-PA | ankyrin repeat domain protein | | |
| CUST_1238_PI406199772 | EE589921.1 | 5.7 | 4.6 |  |  | sg2a salivary protein | |  |
| CUST_13233_PI426302897 | Afun013233 | 5.7 | 4.6 |  | AGAP003295-PA | deoxyribonuclease ii | |  |
| CUST_3220_PI426302897 | Afun003220 | 5.7 | 7.0 |  | AGAP002867-PA | cytochrome p450 | |  |
| CUST_8615_PI426302897 | Afun008615 (CYP6AA1 ) | 5.2 | 3.8 |  | AGAP002862-PA | cytochrome p450 | |  |
| CUST_8965_PI426302897 | Afun008965 | 5.0 | 7.0 |  | AGAP004986-PA | 15 kda selenoprotein | |  |
| CUST_9697_PI426302897 | Afun009697 | 4.8 | 6.8 |  | AGAP006364-PA | abc transporter | |  |
| CUST_9_PI426302915 | CYP6M4.seq | 4.8 | 3.5 |  |  | cytochrome p450 | |  |
| CUST_5559_PI426302897 | Afun005559 | 4.1 | 3.3 |  | AGAP008783-PA | arginase |  |  |
| CUST_13797_PI426302897 | Afun013797 | 4.1 | 3.0 |  | AGAP000289-PA | alcohol dehydrogenase | |  |
| CUST_8445_PI426302897 | Afun008445 (GSTE4) | 3.9 | 4.7 |  | AGAP009193-PA | glutathione-s-transferase gst | | |
| CUST_25_PI426302915 | CYP6Y2_rvcpl.seq | 3.7 | 3.8 |  |  | cytochrome p450 | |  |
| CUST_1392_PI426302897 | Afun001392 | 3.3 | 3.4 |  | NA | glycine dehydrogenase | |  |
| CUST_2908_PI426302897 | Afun002908 | 3.3 | 3.7 |  | NA | ankyrin repeat domain protein | | |
| CUST_3394_PI426302897 | Afun003394 (CYP315A1 ) | 3.1 | 2.4 |  | AGAP000284-PA | cytochrome p450 | |  |
| CUST_8909_PI426302897 | Afun008909 (CYP4K2) | 2.4 | 2.0 |  | AGAP002416-PA | cytochrome p450 | |  |
| CUST_20_PI426302915 | CYP6S1.seq | 2.3 | 2.2 |  |  | cytochrome p450 | |  |
| CUST_7499_PI426302897 | Afun007499 (GSTD1 ) | 2.0 | 2.6 |  | AGAP004164-PA | glutathione transferase | |  |
|  |  |  |  |  |  |  |  |  |
| CUST_2962_PI406199769 | combined_c1493 | 2.8 |  | 2.4 |  | AGAP010698-PA [Anopheles gambiae str. PEST] | | |
| CUST_1400_PI426302897 | Afun001400 | 2.5 |  | 1.9 | AGAP012885-PA | histone cluster h4j-like | |  |
| CUST_1156_PI426302897 | Afun001156 | 2.1 |  | 1.6 | AGAP000609-PA | sg1d salivary protein precursor | | |
|  |  |  |  |  |  |  |  |  |
| CUST_16011_PI406199769 | combined_c8490 | 13.4 |  |  |  | ---NA--- |  |  |
| CUST_7278_PI426302897 | Afun007278 | 12.9 |  |  | AGAP000655-PA | 40s ribosomal protein s14 | |  |
| CUST_2758_PI406199772 | CD578022.1 | 7.7 |  |  |  | ---NA--- |  |  |
| CUST_2215_PI406199769 | combined_c1119 | 7.6 |  |  |  | phospholipid-transporting atpase 1 (aminophospholipid flippase 1) | | |
| CUST_8795_PI406199769 | combined_c4454 | 7.1 |  |  |  | ---NA--- |  |  |
| CUST_1617_PI406199769 | combined_c817 | 6.9 |  |  |  | AGAP003545-PA [Anopheles gambiae str. PEST] | | |
| CUST_1660_PI426302897 | Afun001660 | 6.2 |  |  | NA | conserved hypothetical protein [Culex quinquefasciatus] | | |
| CUST_11942_PI426302897 | Afun011942 | 6.1 |  |  | AGAP011509-PA | carboxylesterase | |  |
| CUST_2892_PI406199769 | combined_c1459 | 5.3 |  |  |  | AGAP005696-PA [Anopheles gambiae str. PEST] | | |
| CUST_4048_PI406199772 | CD577343.1 | 5.3 |  |  |  | cuticle protein | |  |
| CUST_9266_PI406199798 | AGAP006033-RA___2L | 5.1 |  |  | AGAP006033-RA___2L | AGAP006033-PA [Anopheles gambiae str. PEST] | | |
| CUST_12343_PI426302897 | Afun012343 (CYP4H18 ) | 5.0 |  |  | AGAP008358-PA | cytochrome p450 4d1 | |  |
| CUST_13210_PI406199769 | combined_c6758 | 4.9 |  |  |  | ---NA--- |  |  |
| CUST_10836_PI426302897 | Afun010836 | 4.8 |  |  | AGAP006228-PA | esterase b1 |  |  |
| CUST_2641_PI406199769 | combined_c1332 | 4.7 |  |  |  | ---NA--- |  |  |
| CUST_11042_PI426302897 | Afun011042 | 3.9 |  |  | AGAP003321-PA | glycine dehydrogenase | |  |
| CUST_11037_PI426302897 | Afun011037 | 3.7 |  |  | AGAP003581-PA | alcohol dehydrogenase | |  |
| CUST_11779_PI426302897 | Afun011779 | 3.7 |  |  | AGAP003128-PA | conserved hypothetical protein [Culex quinquefasciatus] | | |
| CUST_9750_PI426302897 | Afun009750 | 3.6 |  |  | AGAP001989-PA | secreted salivary gland protein | | |
| CUST_2395_PI426302897 | Afun002395 | 3.3 |  |  | NA | NA |  |  |
| CUST_9113_PI426302897 | Afun009113 | 3.1 |  |  | AGAP000154-PA | amp dependent ligase | |  |
| CUST_1_PI426302915 | CYP6M1a.seq | 2.9 |  |  |  | cytochrome p450 | |  |
| CUST_7501_PI406199798 | AGAP007662-RA___2L | 2.6 |  |  | AGAP007662-RA___2L | short-chain dehydrogenase | | |
| CUST_9335_PI426302897 | Afun009335 (CYP6AG1 ) | 2.4 |  |  | AGAP003343-PA | cytochrome p450 | |  |
| CUST_7769_PI426302897 | Afun007769 (CYP9K1) | 2.2 |  |  | AGAP000818-PA | cytochrome p450 cyp9k1 | |  |
| CUST_11899_PI426302897 | Afun011899 | 2.1 |  |  | AGAP012514-PA | short-chain dehydrogenase | | |
| CUST_8453_PI426302897 | Afun008453 | 2.1 |  |  | AGAP001346-PA | salivary protein | |  |
|  |  |  |  |  |  |  |  |  |
| CUST_982_PI406199772 | EE589254.1 |  |  | 3.5 |  | ge rich salivary gland protein | | |
| CUST_9764_PI426302897 | Afun009764 |  |  | 3.2 | AGAP001863-PB | brain chitinase and chia | |  |
| CUST_1699_PI406199772 | EE589425.1 |  |  | 3.0 |  | d7-related 1 protein | |  |
| CUST_3518_PI406199772 | CD577628.1 |  |  | 3.0 |  | ---NA--- |  |  |
| CUST_379_PI406199772 | EE589823.1 |  |  | 2.0 |  | gsg7 salivary protein | |  |
| CUST_12889_PI426302897 | Afun012889 |  |  | 2.0 | AGAP008302-PA | glycoprotein hormone beta subunit | | |
| CUST_1798_PI406199769 | combined_c908 |  |  | 1.7 |  | ---NA--- |  |  |
|  |  |  |  |  |  |  |  |  |
| CUST_3979_PI406199769 | combined_c2010 |  | 31.3 |  |  | AGAP013493-PA [Anopheles gambiae str. PEST] | | |
| CUST_6802_PI406199769 | combined_c3440 |  | 18.3 |  |  | ---NA--- |  |  |
| CUST_1068_PI426302897 | Afun001068 |  | 16.8 |  | NA | conserved hypothetical protein [Culex quinquefasciatus] | | |
| CUST_1458_PI406199769 | combined_c738 |  | 15.9 |  |  | short-chain dehydrogenase | | |
| CUST_9088_PI426302897 | Afun009088 |  | 9.3 |  | AGAP004900-PA | serine protease | |  |
| CUST_3574_PI426302897 | Afun003574 |  | 6.2 |  | NA | NA |  |  |
| CUST_13835_PI426302897 | Afun013835 |  | 4.5 |  | AGAP003785-PA | choline dehydrogenase | |  |
| CUST_14570_PI426302897 | Afun014570 |  | 4.0 |  | AGAP006400-PA | alkaline phosphatase | |  |
| CUST_4043_PI406199772 | CD577345.1 |  | 3.7 |  |  | cuticle protein | |  |
| CUST_7722_PI426302897 | Afun007722 |  | 3.2 |  | AGAP009850-PA | abc transporter | |  |
| CUST_12261_PI426302897 | Afun012261 |  | 2.8 |  | AGAP005758-PA | carboxylesterase | |  |
| CUST_13481_PI426302897 | Afun013481 (GSTE1 ) |  | 2.7 |  | AGAP009195-PA | glutathione-s-transferase gst | | |
| CUST_8727_PI426302897 | Afun008727 |  | 2.4 |  | AGAP013327-PA | oxidase peroxidase | |  |
| CUST_25_PI406199775 | CYP6P9a |  | 2.3 |  |  | cytochrome p450 | |  |
| CUST_15331_PI426302897 | Afun015331 (CYP307A1) | | 2.2 |  | AGAP001039-PB | cytochrome p450 307a1 | |  |
| CUST_493_PI426302897 | Afun000493 |  | 2.1 |  | AGAP006225-PA | aldehyde oxidase | |  |

**Table S2: The most up-regulated genes in R_DDT_-S, Rperm-S and C-S comparisons**

| **Probe Name** | **Systematic Name** | **R_DDT_-S** | **Rperm-S** | **C-S** | **Ortholog in *An. gambiae*** | **Description** |  |
| --- | --- | --- | --- | --- | --- | --- | --- |
| CUST_10958_PI426302897 | Afun010958 | 143.5 | 30.5 | 77.9 | AGAP012235-PA | prefoldin subunit 6 | |
| CUST_12529_PI426302897 | Afun012529 | 85.6 | 11.7 | 11.5 | AGAP000135-PA | hypothetical protein AaeL_AAEL002059 [Aedes aegypti] | |
| CUST_10990_PI426302897 | Afun010990 | 67.4 | 150.0 | 122.5 | AGAP010693-PA | wd-repeat protein | |
| CUST_11932_PI426302897 | Afun011932 | 62.9 | 57.2 | 5.9 | AGAP002875-PA | AGAP002875-PA [Anopheles gambiae str. PEST] | |
| CUST_8968_PI426302897 | Afun008968 | 62.5 | 39.8 | 4.9 | AGAP011375-PA | selenophosphate synthase | |
| CUST_4860_PI426302897 | Afun004860 | 44.4 | 33.4 | 51.7 | NA | NA |  |
| CUST_500_PI426302897 | Afun000500 | 36.2 | 38.1 | 41.4 | NA | glycogenin |  |
| CUST_12565_PI426302897 | Afun012565 | 30.0 | 33.7 | 38.0 | AGAP011220-PA | AGAP011220-PA [Anopheles gambiae str. PEST] | |
| CUST_12810_PI426302897 | Afun012810 | 30.7 | 17.2 | 22.0 | AGAP001428-PA | AGAP001428-PA [Anopheles gambiae str. PEST] | |
| CUST_6391_PI426302897 | Afun006391 | 25.8 | 48.9 | 55.7 | AGAP010956-PA | AGAP010956-PA [Anopheles gambiae str. PEST] | |
| CUST_9227_PI426302897 | Afun009227 | 22.2 | 23.3 | 25.6 | AGAP008141-PA | argininosuccinate lyase | |
| CUST_2403_PI426302897 | Afun002403 | 21.8 | 28.7 | 33.1 | NA | conserved hypothetical protein [Culex quinquefasciatus] | |
| CUST_15344_PI426302897 | Afun015344 | 13.5 | 12.9 | 12.0 | AGAP005574-PA | zinc finger protein 343 | |
| CUST_45_PI426302897 | Afun000045 (GSTE2) | 13.3 | 10.9 | 15.2 | AGAP009194-PA | glutathione-s-transferase gst | |
| CUST_4223_PI426302897 | Afun004223 | 12.8 | 10.6 | 9.4 | AGAP008358-PA | cytochrome p450 4d1 | |
| CUST_9697_PI426302897 | Afun009697 | 11.6 | 4.8 | 6.8 | AGAP006364-PA | abc transporter | |
| CUST_1822_PI406199769 | combined_c920 | 11.5 | 11.1 | 11.4 |  | glutathione-s-transferase gst | |
| CUST_8360_PI426302897 | Afun008360 | 11.2 | 8.7 | 12.3 | AGAP000183-PA | AGAP000183-PA [Anopheles gambiae str. PEST] | |
| CUST_3977_PI426302897 | Afun003977 | 11.0 | 11.2 | 10.8 | NA | NA |  |
| CUST_3220_PI426302897 | Afun003220 | 10.8 | 5.7 | 7.0 | AGAP002867-PA | cytochrome p450 | |
| CUST_1894_PI426302897 | Afun001894 | 9.7 | 14.4 | 4.7 | AGAP000154-PA | amp dependent ligase | |
| CUST_4529_PI426302897 | Afun004529 | 8.2 | 18.6 | 22.1 | AGAP012173-PA | ankyrin repeat domain protein | |
| CUST_8237_PI426302897 | Afun008237 | 8.1 | 9.2 | 7.7 | AGAP010698-PA | hypothetical protein AaeL_AAEL003949 [Aedes aegypti] | |
| CUST_9492_PI426302897 | Afun009492 | 7.9 | 16.4 | 3.7 | AGAP001722-PA | carboxylesterase | |
| CUST_8965_PI426302897 | Afun008965 | 6.3 | 5.0 | 7.0 | AGAP004986-PA | 15 kda selenoprotein | |
| CUST_14076_PI426302897 | Afun014076 | 5.9 | 8.3 | 15.5 | AGAP000603-PA | AGAP000603-PA [Anopheles gambiae str. PEST] | |
| CUST_10777_PI426302897 | Afun010777 | 5.8 | 6.5 | 4.6 | AGAP004091-PA | 28s ribosomal protein s5 | |
| CUST_8615_PI426302897 | Afun008615 (CYP6AA1 ) | 5.1 | 5.2 | 3.8 | AGAP002862-PA | cytochrome p450 | |
| CUST_9_PI426302915 | CYP6M4.seq | 4.9 | 4.8 | 3.5 |  | cytochrome p450 | |
| CUST_8445_PI426302897 | Afun008445 (GSTE4) | 4.3 | 3.9 | 4.7 | AGAP009193-PA | glutathione-s-transferase gst | |
| CUST_4200_PI406199772 | CD577267.1 | 4.3 | 2.6 | 2.6 |  | cytochrome b | |
| CUST_6930_PI426302897 | Afun006930 (CYP6M7) | 4.0 | 34.6 | 28.9 | AGAP008212-PA | cytochrome p450 6a8 | |
| CUST_5559_PI426302897 | Afun005559 | 3.5 | 4.1 | 3.3 | AGAP008783-PA | arginase |  |
| CUST_25_PI426302915 | CYP6Y2_rvcpl.seq | 3.4 | 3.7 | 3.8 |  | cytochrome p450 | |
| CUST_20_PI426302915 | CYP6S1.seq | 3.0 | 2.3 | 2.2 |  | cytochrome p450 | |
| CUST_4337_PI426302897 | Afun004337 | 3.0 | 3.0 | 3.5 | AGAP011798-PA | acyl- oxidase | |
| CUST_8909_PI426302897 | Afun008909 (CYP4K2) | 2.8 | 2.4 | 2.0 | AGAP002416-PA | cytochrome p450 | |
| CUST_1392_PI426302897 | Afun001392 | 2.5 | 3.3 | 3.4 | NA | glycine dehydrogenase | |
| CUST_13218_PI426302897 | Afun013218 (CYP315A1) | 2.3 | 2.9 | 2.6 | AGAP000284-PA | cytochrome p450 | |
| CUST_7499_PI426302897 | Afun007499 (GSTD1) | 2.2 | 2.0 | 2.6 | AGAP004164-PA | glutathione transferase | |
|  |  |  |  |  |  |  |  |
| CUST_4069_PI426302897 | Afun004069 | 18.9 | 23.3 |  | AGAP006275-PA | hemolymph protein | |
| CUST_9232_PI426302897 | Afun009232 | 11.5 | 4.0 |  | AGAP004236-PA | AGAP004236-PA [Anopheles gambiae str. PEST] | |
| CUST_10767_PI406199769 | combined_c5462 | 7.8 | 5.7 |  |  | ---NA--- |  |
| CUST_10030_PI406199769 | combined_c5082 | 7.2 | 2.4 |  |  | ---NA--- |  |
| CUST_11037_PI426302897 | Afun011037 | 6.5 | 3.7 |  | AGAP003581-PA | alcohol dehydrogenase | |
| CUST_9266_PI406199798 | AGAP006033-RA___2L | 6.2 | 5.1 |  | AGAP006033-RA___2L | AGAP006033-PA [Anopheles gambiae str. PEST] | |
| CUST_3860_PI406199769 | combined_c1950 | 5.8 | 2.9 |  |  | ---NA--- |  |
| CUST_12343_PI426302897 | Afun012343 (CYP4H18 ) | 5.2 | 5.0 |  | AGAP008358-PA | cytochrome p450 4d1 | |
| CUST_2445_PI406199772 | CD578178.1 | 5.1 | 6.7 |  |  | ---NA--- |  |
| CUST_10836_PI426302897 | Afun010836 | 4.3 | 4.8 |  | AGAP006228-PA | esterase b1 |  |
| CUST_10986_PI426302897 | Afun010986 | 4.0 | 4.8 |  | AGAP003707-PA | elof1_drome ame: full=transcription elongation factor 1 homolog | |
| CUST_7769_PI426302897 | Afun007769 (CYP9K1 ) | 3.0 | 2.2 |  | AGAP000818-PA | cytochrome p450 cyp9k1 | |
| CUST_13137_PI426302897 | Afun013137 | 3.0 | 2.1 |  | AGAP001704-PA | isopentenyl pyrophosphate:dimethylallyl pyrophosphate isomerase | |
| CUST_8045_PI426302897 | Afun008045 | 3.0 | 2.8 |  | AGAP000260-PB | atp synthase subunit mitochondrial | |
| CUST_7469_PI426302897 | Afun007469 (CYP9J3) | 2.0 | 2.1 |  | AGAP012296-PA | cytochrome p450 | |
|  |  |  |  |  |  |  |  |
| CUST_3346_PI406199769 | combined_c1691 |  | 20.4 | 20.4 |  | mediator complex | |
| CUST_14204_PI426302897 | Afun014204 |  | 19.1 | 3.8 | AGAP004911-PA | AGAP004911-PA [Anopheles gambiae str. PEST] | |
| CUST_12461_PI426302897 | Afun012461 |  | 8.4 | 10.9 | AGAP000288-PA | alcohol dehydrogenase | |
| CUST_12651_PI426302897 | Afun012651 |  | 8.1 | 9.2 | AGAP005769-PA | cg16865 cg16865-pa | |
| CUST_5545_PI426302897 | Afun005545 |  | 5.7 | 4.9 | AGAP012173-PA | ankyrin repeat domain protein | |
| CUST_1238_PI406199772 | EE589921.1 |  | 5.7 | 4.6 |  | sg2a salivary protein | |
| CUST_4064_PI426302897 | Afun004064 |  | 5.6 | 3.4 | AGAP003205-PA | monocarboxylate transporter | |
| CUST_9290_PI426302897 | Afun009290 |  | 5.3 | 3.3 | AGAP007851-PA | kda salivary secreted protein | |
| CUST_12219_PI406199769 | combined_c6219 |  | 4.7 | 5.7 |  | ---NA--- |  |
| CUST_12763_PI426302897 | Afun012763 |  | 4.6 | 8.4 | AGAP003410-PA | AGAP003410-PA [Anopheles gambiae str. PEST] | |
| CUST_7859_PI406199769 | combined_c3979 |  | 4.3 | 4.2 |  | ---NA--- |  |
| CUST_3248_PI406199769 | combined_c1641 |  | 2.8 | 3.0 |  | kda salivary protein | |
| CUST_8013_PI426302897 | Afun008013 |  | 2.5 | 2.3 | AGAP009783-PA | acyl-coa dehydrogenase | |
| CUST_9838_PI426302897 | Afun009838 |  | 2.2 | 3.2 | AGAP004859-PA | serine protease | |
| CUST_3489_PI406199769 | combined_c1762 |  | 2.2 | 2.2 |  | abc transporter | |
| CUST_8026_PI426302897 | Afun008026 |  | 2.1 | 2.2 | AGAP003578-PA | aldehyde dehydrogenase | |
| CUST_13685_PI406199769 | combined_c7027 |  | 2.09 | 4.9 |  | ---NA--- |  |
|  |  |  |  |  |  |  |  |
| CUST_9157_PI426302897 | Afun009157 | 78.6 |  | 73.6 | AGAP009075-PA | dead box atp-dependent rna helicase | |
| CUST_6802_PI406199769 | combined_c3440 | 65.0 |  | 18.3 |  | ---NA--- |  |
| CUST_6803_PI406199769 | combined_c3440 | 36.6 |  | 23.4 |  | ---NA--- |  |
| CUST_3979_PI406199769 | combined_c2010 | 21.2 |  | 31.3 |  | AGAP013493-PA [Anopheles gambiae str. PEST] | |
| CUST_1458_PI406199769 | combined_c738 | 10.4 |  | 15.9 |  | short-chain dehydrogenase | |
| CUST_9088_PI426302897 | Afun009088 | 9.1 |  | 9.3 | AGAP004900-PA | serine protease | |
| CUST_1623_PI426302897 | Afun001623 | 8.3 |  | 13.3 | AGAP003918-PA | AGAP003918-PA [Anopheles gambiae str. PEST] | |
| CUST_9312_PI426302897 | Afun009312 | 8.0 |  | 10.1 | AGAP009768-PA | af141930_1high affinity gaba transporter | |
| CUST_14790_PI426302897 | Afun014790 | 6.2 |  | 7.1 | AGAP002448-PA | cysteine-rich venom | |
| CUST_14570_PI426302897 | Afun014570 | 5.3 |  | 4.0 | AGAP006400-PA | alkaline phosphatase | |
| CUST_4043_PI406199772 | CD577345.1 | 4.4 |  | 3.7 |  | cuticle protein | |
| CUST_15331_PI426302897 | Afun015331 (CYP307A1) | 3.4 |  | 2.2 | AGAP001039-PB | cytochrome p450 307a1 | |
| CUST_25_PI406199775 | CYP6P9a | 2.8 |  | 2.3 |  | cytochrome p450 | |
| CUST_13481_PI426302897 | Afun013481 (GSTE1 ) | 2.5 |  | 2.7 | AGAP009195-PA | glutathione-s-transferase gst | |
|  |  |  |  |  |  |  |  |
| CUST_16011_PI406199769 | combined_c8490 |  | 13.4 |  |  | ---NA--- |  |
| CUST_7278_PI426302897 | Afun007278 |  | 12.9 |  | AGAP000655-PA | 40s ribosomal protein s14 | |
| CUST_5112_PI406199769 | combined_c2590 |  | 8.2 |  |  | ---NA--- |  |
| CUST_1617_PI406199769 | combined_c817 |  | 6.9 |  |  | AGAP003545-PA [Anopheles gambiae str. PEST] | |
| CUST_1660_PI426302897 | Afun001660 |  | 6.2 |  | NA | conserved hypothetical protein [Culex quinquefasciatus] | |
| CUST_2892_PI406199769 | combined_c1459 |  | 5.3 |  |  | AGAP005696-PA [Anopheles gambiae str. PEST] | |
| CUST_4048_PI406199772 | CD577343.1 |  | 5.3 |  |  | cuticle protein | |
| CUST_11042_PI426302897 | Afun011042 |  | 3.9 |  | AGAP003321-PA | glycine dehydrogenase | |
| CUST_7081_PI426302897 | Afun007081 |  | 3.3 |  | AGAP004152-PA | AGAP004152-PA [Anopheles gambiae str. PEST] | |
| CUST_1_PI426302915 | CYP6M1a.seq |  | 2.9 |  |  | cytochrome p450 | |
| CUST_9335_PI426302897 | Afun009335 (CYP6AG1) |  | 2.4 |  | AGAP003343-PA | cytochrome p450 | |
| CUST_14653_PI426302897 | Afun014653 |  | 2.3 |  | AGAP003494-PA | sugar transporter | |
| CUST_3249_PI406199769 | combined_c1641 |  | 2.3 |  |  | kda salivary protein | |
|  |  |  |  |  |  |  |  |
| CUST_3508_PI406199772 | CD577633.1 | 6.2 |  |  |  | ---NA--- |  |
| CUST_10818_PI426302897 | Afun010818 | 5.5 |  |  | AGAP011626-PA | mediator complex | |
| CUST_6620_PI426302897 | Afun006620 | 4.3 |  |  | AGAP003582-PA | sorbitol dehydrogenase | |
| CUST_8151_PI406199769 | combined_c4126 | 4.1 |  |  |  | gag-protease-integrase-rt-r polyprotein | |
| CUST_1782_PI406199772 | EE589336.1 | 3.8 |  |  |  | sg2a salivary protein | |
| CUST_9746_PI406199769 | combined_c4936 | 3.7 |  |  |  | reverse transcriptase | |
| CUST_1416_PI406199769 | combined_c717 | 3.5 |  |  |  | AGAP007557-PA [Anopheles gambiae str. PEST] | |
| CUST_13475_PI426302897 | Afun013475 | 3.2 |  |  | AGAP003582-PA | alcohol dehydrogenase | |
| CUST_5448_PI426302897 | Afun005448 (CYP302A1) | 2.6 |  |  | AGAP005992-PA | cytochrome p450 | |
| CUST_7403_PI426302897 | Afun007403 | 2.5 |  |  | AGAP003785-PC | glucose dehydrogenase | |
| CUST_4047_PI406199772 | CD577343.1 | 2.4 |  |  |  | cuticle protein | |
| CUST_8823_PI426302897 | Afun008823 (CYP4D15 ) | 2.4 |  |  | AGAP002418-PA | cytochrome p450 | |
| CUST_7301_PI426302897 | Afun007301 (CYP4J5) | 2.2 |  |  | AGAP006048-PA | cytochrome p450 | |
| CUST_208_PI406199788 | gb-CYP12F3 | 2.1 |  |  |  | cytochrome p450 | |
| CUST_10630_PI426302897 | Afun010630 | 2.1 |  |  | AGAP002866-PA | cytochrome p450 | |
|  |  |  |  |  |  |  |  |
| CUST_1068_PI426302897 | Afun001068 |  |  | 16.8 | NA | conserved hypothetical protein [Culex quinquefasciatus] | |
| CUST_9477_PI426302897 | Afun009477 |  |  | 5.1 | AGAP012173-PA | ankyrin repeat domain protein | |
| CUST_12046_PI426302897 | Afun012046 |  |  | 4.9 | AGAP002604-PA | hypothetical conserved protein | |
| CUST_9340_PI426302897 | Afun009340 |  |  | 4.5 | AGAP012837-PA | 39s ribosomal protein l43 | |
| CUST_13835_PI426302897 | Afun013835 |  |  | 4.5 | AGAP003785-PA | choline dehydrogenase | |
| CUST_10703_PI426302897 | Afun010703 |  |  | 4.1 | AGAP001073-PC | cg12236- isoform a | |
| CUST_7722_PI426302897 | Afun007722 |  |  | 3.2 | AGAP009850-PA | abc transporter | |
| CUST_12261_PI426302897 | Afun012261 |  |  | 2.8 | AGAP005758-PA | carboxylesterase | |
| CUST_4088_PI406199772 | CD577323.1 |  |  | 2.4 |  | cuticle protein | |
| CUST_493_PI426302897 | Afun000493 |  |  | 2.1 | AGAP006225-PA | aldehyde oxidase |  |
